# KaiWei JianPI Ointment Downregulates Fcgbp and Modulates HMGB1/TLR4 Signaling to Combat Ferroptosis in *H. pylori* Infection

**DOI:** 10.1155/cjid/4297059

**Published:** 2026-04-18

**Authors:** Xiaoying Zhu, Jie Liu, Meng Chen, Yubo Jin, Yan Dang, Xianfeng Qin, Huijun Tang, Caiqun Bie

**Affiliations:** ^1^ Shenzhen Clinical College of Integrated Chinese and Western Medicine, Guangzhou University of Chinese Medicine, Guangzhou, China, gzucm.edu.cn; ^2^ Gastroenterology Department, Shenzhen Hospital of Integrated Traditional Chinese and Western Medicine, Shenzhen, China; ^3^ Guangdong Key Laboratory for Research and Development of Natural Drugs, Guangdong Medical University, Dongguan, China, gdmu.edu.cn

**Keywords:** Fcgbp, ferroptosis, *H. pylori* infection, KaiWei JianPI ointment, TLR4/HMGB1

## Abstract

**Objective:**

This study aimed to explore the therapeutic mechanisms of KaiWei JianPI Ointment in treating *H. pylori* infection, focusing on its role in regulating ferroptosis and the associated HMGB1/TLR4 signaling pathway.

**Methods:**

We used a combination of network pharmacology, molecular docking, transcriptome sequencing, and molecular biology in this study. Active components of the KaiWei JianPI Ointments were identified using the Traditional Chinese Medicine Systems Pharmacology database. Post‐treatment transcriptomic changes in rat gastric tissues were analyzed to identify differentially expressed genes.

**Results:**

Network analysis identified 152 active components targeting key proteins involved in immune responses and cellular signaling. Transcriptome sequencing revealed 76 differentially expressed genes linked to immune response pathways. Additionally, treatment with the KaiWei JianPI Ointment resulted in decreased levels of Fe^2+^ and MDA in *H. pylori*‐infected rats, suggesting inhibition of ferroptosis. This effect correlated with the downregulation of Fcgbp and modulation of HMGB1/TLR4 signaling.

**Conclusions:**

KaiWei JianPI Ointment shows potential as a novel therapeutic strategy against *H. pylori* infection by inhibiting ferroptosis through modulation of the HMGB1/TLR4 pathway. This study provides insights into the application of traditional Chinese medicine in modern treatment frameworks, highlighting the need for clinical validation and further exploration of the underlying mechanisms.

## 1. Introduction

*Helicobacter pylori* (*H. pylori*) is a Gram‐negative bacterium that plays a critical role in the development of chronic gastritis, peptic ulcers, and gastric cancer [1]. Humans are particularly susceptible to *H. pylori* infection, serving as its sole natural hosts. Primary transmission occurs through “oral‐oral” and “fecal‐oral” routes, with household transmission being a significant factor for both new infections and reinfections [2]. *H. pylori* is the sole identified pathogen with a proven causal link to the development of gastric cancer in humans and is classified as a Group I carcinogen [3]. A substantial proportion of gastric cancer cases are associated with *H. pylori* infection. The global prevalence of *H. pylori* exceeds 50% [4], with even higher prevalence rates in developing countries. In China, the infection rate is reported to be 44.2% [5].

Consensus guidelines increasingly recommend therapies for the eradication of *H. pylori* infection in individuals. However, the global prevalence of antibiotic resistance has contributed to a high rate of treatment failure, undermining clinical efficacy [6]. Significant resistance has been documented against commonly prescribed antibiotics, such as metronidazole, clarithromycin, and levofloxacin [7], which has led to unsatisfactory eradication rates and necessitated revisions to treatment protocols. Unfortunately, as the use of antibiotics increases, *H. pylori* resistance continues to rise each year, resulting in refractory *Helicobacter pylori* infection (RHPI), which has emerged as a pressing clinical challenge requiring urgent attention.

Ferroptosis is a form of regulated cell death characterized by iron‐dependent lipid peroxidation. Proposed in 2012, this process has been extensively investigated for its involvement in a range of physiological and pathological conditions [8]. *H. pylori* infection substantially affects processes associated with ferroptosis; specifically, *H. pylori* and its components reduce the expression of LPCAT3, an enzyme involved in generating lipid peroxidation substrates. Additionally, these components downregulate genes associated with iron uptake, such as TfR1, while counteracting glutathione depletion by upregulating the cystine/glutamate reverse transporter subunit SLC3A2, ultimately mitigating ferroptosis [9]. *H. pylori* infection may promote gastric cancer by influencing ferroptosis‐related genes. The ferroptosis‐related gene SOCS1 is a potential prognostic biomarker and is significantly upregulated in patients with *H. pylori* infection and gastric adenocarcinoma (STAD), which may correlate with tumor immune infiltration [10]. Additionally, Liu et al. found that YWHAE was highly expressed in *H. pylori*‐related gastritis and gastric cancer, positively correlating with ferroptosis and involving multiple cancer signaling pathways [11]. Zhu et al. reported that *H. pylori* infection increased gastric cancer cell sensitivity to RSL3‐induced ferroptosis, suggesting an association between ferroptosis‐related genes and the tumor microenvironment [12]. Thus, *H. pylori* appears to influence the regulation and facilitation of ferroptosis through multiple pathways.

Toll‐like receptors (TLRs) serve as essential recognition receptors that play a crucial role in innate immunity by aiding immune cells in recognizing microbial antigens. TLRs expressed on the surface of gastric epithelial cells (TLRs 1, 2, 4, 5, 6, and 10) and intracellular TLR9 interact with *H. pylori* virulence factors, activate the nuclear factor kappa‐light‐chain‐enhancer of activated B cells (NF‐κB), and promote the release of proinflammatory cytokines [13]. TLR polymorphisms may affect host susceptibility to *H. pylori* infection and modulate cytokine responses related to gastritis and immune activation. For example, TLR4 polymorphisms are linked to an elevated risk of *H. pylori* infection and a diminished risk of chronic atrophic gastritis and intestinal metaplasia in Chinese patients [14]. Castano‐Rodriguez et al. identified six TLR4 polymorphisms that were significantly associated with gastric cancer [15]. High‐mobility group box 1 (HMGB1) acts as an endogenous ligand for TLR4 and activates inflammatory responses via TLR4 signaling [16]. The release of HMGB1 is also correlated with *H. pylori* infection, exacerbating inflammatory responses [17]. Moreover, the HMGB1/TLR4 signaling pathway plays a crucial role in regulating ferroptosis and associated inflammatory responses, leading to tissue damage.

Traditional Chinese medicine (TCM) classifies *H. pylori* as a pathogenic factor associated with damp‐heat and pathogenic qi [18]. The primary pathogenic mechanisms involve the disharmony of stomach qi and reversed qi flow, with phlegm, dampness, and stasis identified as pathological products. Treatment principles focus on strengthening the spleen, eliminating dampness, and soothing the liver and stomach to restore the transportation, reception, and ascending‐descending functions of the middle burner. Clinical studies indicate that TCM offers unique advantages in the treatment of *H. pylori* infections. The KaiWei JianPI Ointment, based on a modified Shenling Baizhu San formula, demonstrated significant clinical efficacy in *H. pylori* eradication. Network pharmacology analysis reveals its anti‐inflammatory and immunomodulatory properties, while modern pharmacological studies confirm the anti‐inflammatory and antioxidant effects of various herbs in the ointment [11]. Furthermore, our previous retrospective clinical study supports the improvements attributed to KaiWei JianPI Ointment.

In this study, we investigated the therapeutic mechanisms of the KaiWei JianPI Ointment against *H. pylori* infection using network pharmacology, molecular docking, transcriptome sequencing, and molecular biology techniques. Our findings suggest that KaiWei JianPI Ointment may downregulate the Fc fragment of IgG binding protein (Fcgbp) to modulate the HMGB1/TLR4 signaling pathway, providing a potential therapeutic target for *H. pylori*‐related gastric mucosal diseases.

## 2. Materials and Methods

### 2.1. Cyberpharmacology

The ingredients and indications of KaiWei JianPI Ointment were obtained from the TCMSP database. Active ingredients were chosen based on their extent of absorption in the body and similarity to commercial drugs. The UniProt database was used for predictions, and disease‐related targets were obtained from Gene, DrugBank, OMIM, and PharmGKB. A protein–protein interaction (PPI) network was constructed using Cytoscape version 3.10.1. Additionally, gene ontology (GO) and KEGG pathway enrichment analyses were performed on the overlapping targets.

### 2.2. Transcriptome Sequencing

Rat gastric tissue samples were lysed and purified for extraction. mRNA was isolated and subjected to cDNA synthesis and library preparation. Sequencing was performed using DNBSEQ technology and filtered for quality. Differential gene expression analysis identified dysregulated genes; subsequent GO and KEGG analyses were conducted to explore biological implications.

### 2.3. Animal Experimentation

The animal experiments conducted in this study strictly adhered to the ethical standards established by Peking University, The Hong Kong University of Science and Technology and Shenzhen Medical. The study was granted ethical approval with the number [2024‐574], dated [2024‐7‐05]. Throughout the experiments, we strictly followed the ethical guidelines for animal research. Adult male rats (35), weighing between 200 and 250 g, were sourced from Guangzhou Saibainuo Biotechnology Co., Ltd. *H. pylori* Sydney strain 1 (SSI), known to express CagA and VacA, was used in the study. The experimental design incorporated quadruple therapy with omeprazole, amoxicillin, clarithromycin, and pectin along with a spleen‐tonifying herbal paste. Following a 24‐h fast, the model group (30 rats) received 1.5 mL of *H. pylori* suspension (1 × 10^9^ CFU/mL) on alternate days for a total of three administrations, while the normal group (5 rats) received saline. After 2 weeks, three rats from the model group were euthanized for gastric tissue collection and examined using various staining methods to confirm successful colonization. Postmodeling, rats were categorized into treatment groups, receiving specific doses of quadruple therapy and herbal paste, or saline.

### 2.4. Staining Procedures

Warthin–Starry silver staining, immunohistochemistry, Giemsa staining, and hematoxylin and eosin (H&E) staining were performed to confirm *H. pylori* infection in gastric tissues.

### 2.5. Detection of Fe^2+^

Iron Colorimetric Assay Kit (#I291, DOJINDO LABORATORIES) was used to measure Fe^2+^ levels in gastric tissue samples. The tissue (100 mg) was mixed with 1 mL of cold PBS, vortexed, and centrifuged at 14,000 × g for 4 min. The pellet was ground in liquid nitrogen and mixed with 1.3 mL of buffer. The mixture was sonicated for 5 min, cooled, and centrifuged at 160,00 × g for 10 min; and then incubated at 37°C for 15 min, placed in a 96‐well plate. After 60 min, absorbance was measured at 593 nm to determine Fe^2+^ concentration.

### 2.6. Malondialdehyde (MDA) Assay

MDA levels were measured using a Lipid Peroxidation MDA Assay Kit (Catalog #S0053, Beyotime) according to the manufacturer’s instructions.

### 2.7. GSH and GSSG Detection

GSH and GSSG levels were quantified using a GSH/GSSG Kit (Catalog #S0131S, Beyotime). The tissue (10 mg) was homogenized in 100 μL of reagent, cooled for 10 min, and centrifuged at 10,000 × g for 10 min. The supernatant was diluted for subsequent GSH and GSSG measurements.

### 2.8. Western Blot Analysis

Rat stomach tissues were treated with RIPA buffer and protease inhibitors. Homogenized proteins were placed in EP tubes and incubated with antibodies (FCGBP #bs‐13168R from Bioss, GPX4 #ab125066 from Abcam, HMGB1 #82973‐1‐RR from Proteintech, and TLR4#19811‐1‐AP from Proteintech) for 1 h at room temperature. Luminescent imaging was performed using a special solution from Millipore.

### 2.9. Real‐Time PCR

RNA was extracted from rat gastric tissue using a Promega kit. Trizol was added to the samples, which were then lysed at 0°C for 30 min. An equal volume of ethanol was added to the mixture and centrifuged. RNA was purified and eluted with water; purity and concentration of the extracted RNA were determined using a NanoDrop Lite spectrophotometer. cDNA was synthesized using the Evo M‐MLV Reverse Transcriptase Premix. SYBR Green Premix Pro Taq HS was used for the qPCR analysis. Gene expression levels were determined by analyzing qPCR data. Gene‐specific primers used in this study are detailed in Table 1.

**TABLE 1:** Primer sequences for qPCR experiment.

Gene name	Species	Direction	Primer sequences
Fcgbp	Rat	Forward	TGG​CAC​AAT​GGC​ACC​CTC​AAT​AC
		Reverse	CAT​ACT​CCT​CCA​CTC​GGT​CCT​CTG

HMGB1	Rat	Forward	CAA​AGG​CTG​ACA​AGG​CTC​GTT​ATG
		Reverse	GCG​GTA​CTC​AGA​ACA​GAA​CAA​GAA​G

TLR4	Rat	Forward	CGC​TCT​GGC​ATC​ATC​TTC​ATT​GTC
		Reverse	CCT​CCC​ATT​CCA​GGT​AGG​TGT​TTC

β‐actin	Rat	Forward	GCT​GTG​CTA​TGT​TGC​CCT​AGA​CTT​C
		Reverse	GGA​ACC​GCT​CAT​TGC​CGA​TAG​TG

### 2.10. Statistical Analysis

Data are presented as mean ± SEM. IBM SPSS and GraphPad Prism software were used for data analysis. The statistical tests used were the *t*‐test and one‐way ANOVA. Statistical significance was set at *p* < 0.05.

## 3. Results

The network pharmacology study of KaiWei JianPI Ointment identified 152 active components from the TCMSP database, filtered by oral bioavailability (OB ≥ 30%) and drug‐likeness (DL ≥ 0.18). Target prediction yielded 2326 potential targets with 88 unique genes after removing duplicates. Using the keyword “*Helicobacter pylori* Infection” across GeneCards, DrugBank, OMIM, and PharmGKB databases, a dataset of 2578 disease‐related targets was compiled. Mapping these targets with drug targets resulted in 152 predicted targets (Figures 1(a) and 1(b)).

FIGURE 1Network topology study of the gastric tonic in treating *H. pylori* infection. (a, b) Mapping of disease and drug targets. (c) Drug‐component‐target‐disease network diagram. (d–f) Analysis of the core protein–protein interaction (PPI) network. (g) Bar chart of gene ontology (GO) enrichment analysis. (h) Bubble chart of KEGG pathway analysis. (i) Mechanistic diagram of gastric tonic treatment for *H. pylori* infection. (j) Visualization of molecular docking between components and critical targets.

**Figure:**
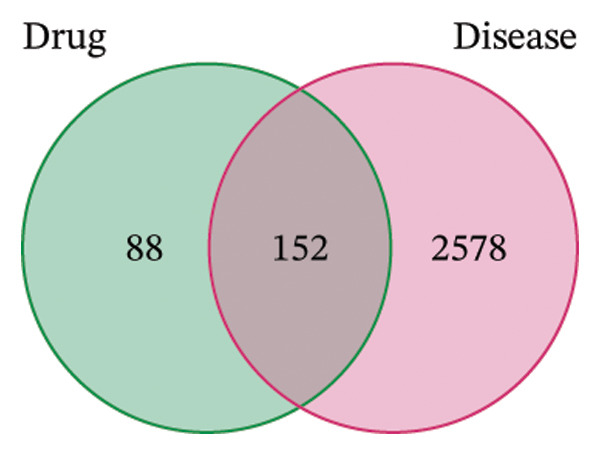
(a)

**Figure:**
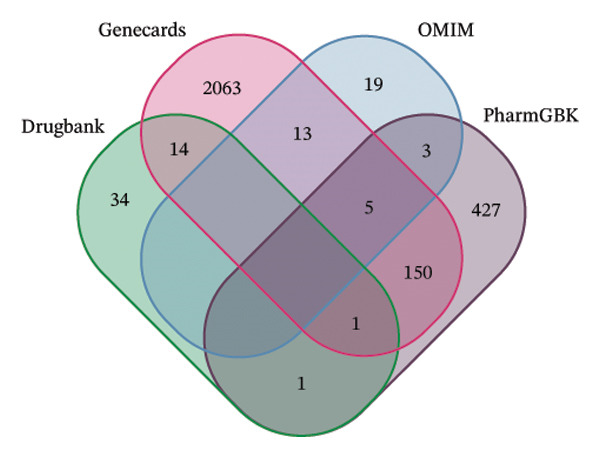
(b)

**Figure:**
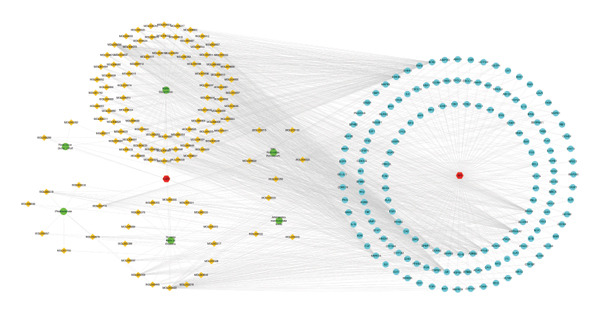
(c)

**Figure:**
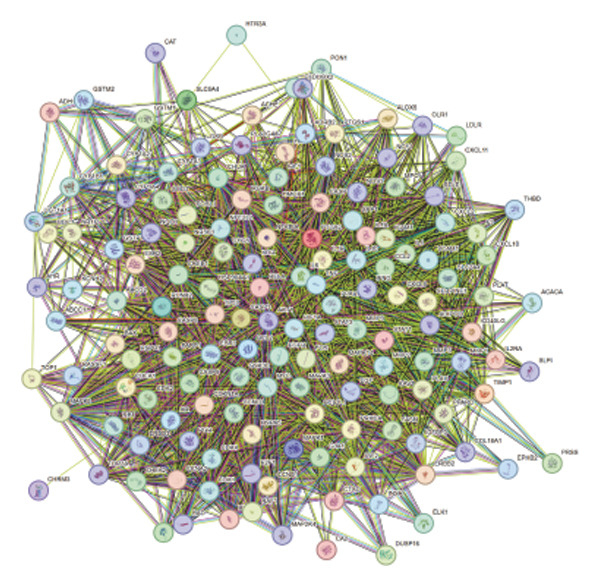
(d)

**Figure:**
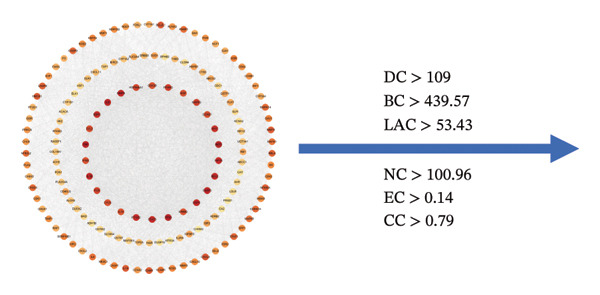
(e)

**Figure:**
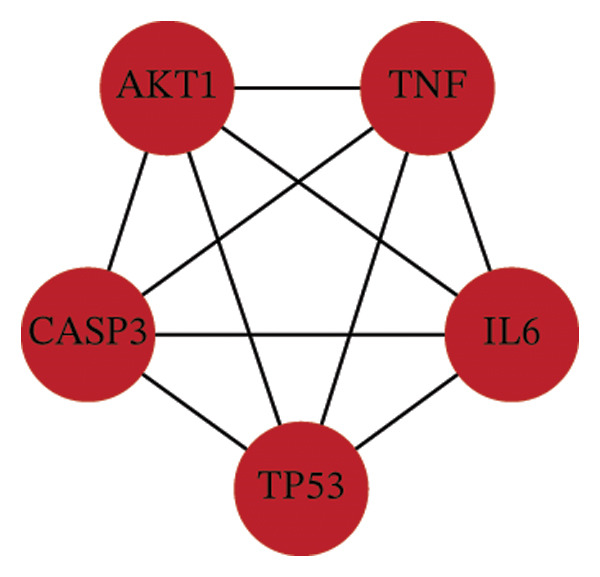
(f)

**Figure:**
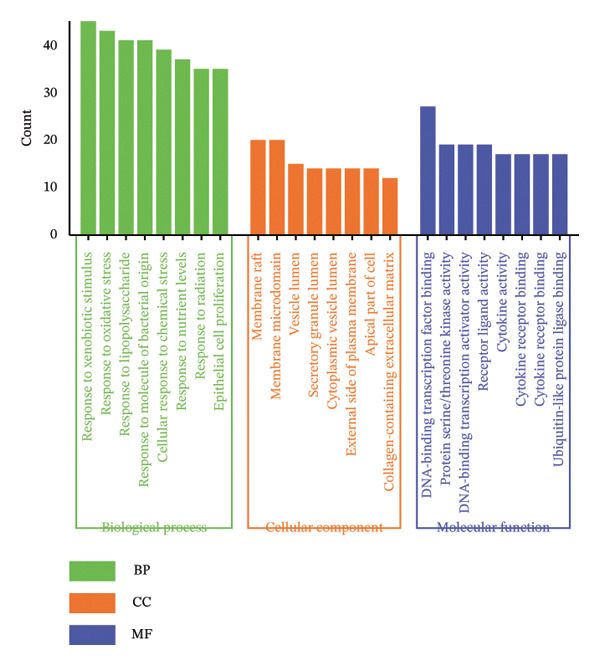
(g)

**Figure:**
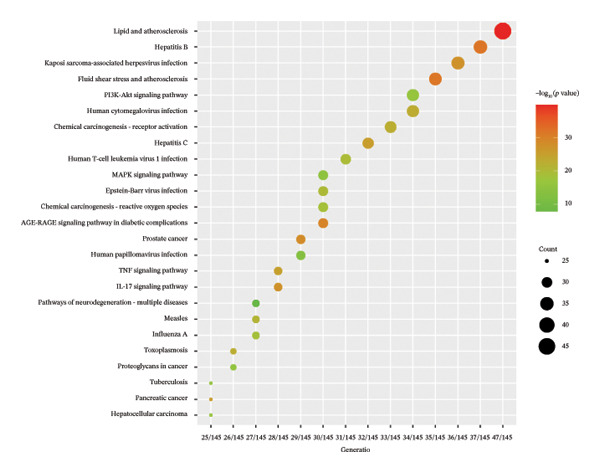
(h)

**Figure:**
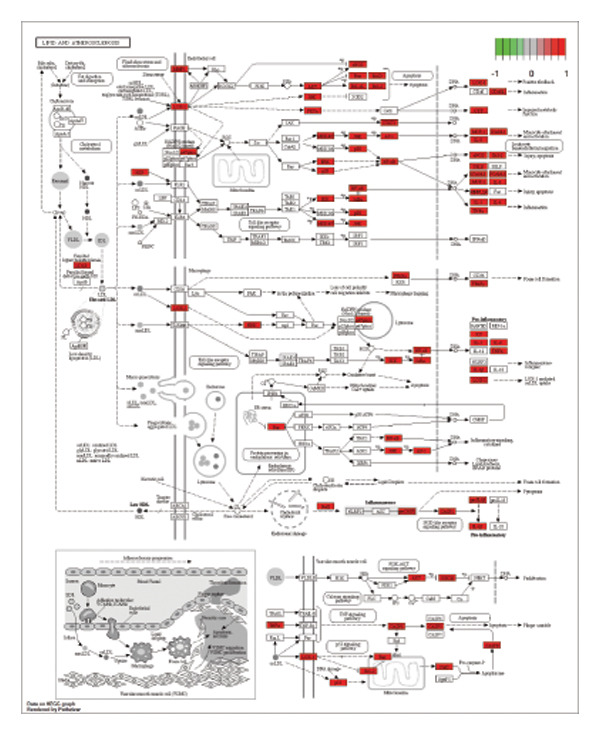
(i)

**Figure:**
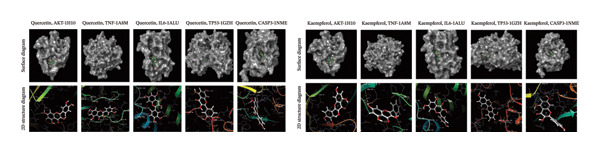
(j)

Analysis of the 152 targets in the STRING database generated a PPI network, which was further examined using Cytoscape for network topology. The top five proteins, AKT1, TNF‐, CASP3, IL6, and TP53, were identified based on degree centrality (DC > 109), betweenness centrality (BC > 439.57), and other metrics (Figures 1(d), 1(e), and 1(f)).

GO and KEGG pathway enrichment analyses using the DAVID platform revealed 2586 GO terms (*p* < 0.05). The significant biological processes included responses to xenobiotic stimuli and lipopolysaccharides and oxidative stress (Figure 1(g)). KEGG identified 181 significant pathways (*p* < 0.05), notably lipid and atherosclerosis, hepatitis B, and human cytomegalovirus infection pathways, suggesting that these may be critical pathways for the KaiWei JianPIOintment treatment of *H. pylori* infection (Figure 1(h)).

The “drug‐component‐target‐disease” network constructed in Cytoscape illustrated the relationships among the 152 targets (Figure 1(c)). Key active compounds identified were quercetin and kaempferol, which targeted AKT1, TNF, CASP3, IL6, and TP53. Molecular docking showed favorable binding affinities, indicating that the active components of KaiWei JianPI Ointment interact effectively with *H. pylori*‐related targets, with an average binding energy of −5.33 kcal/mol (Figure 1(j)).

### 3.1. Transcriptome Analysis of KaiWei JianPI Ointment‐Treatment of *H. pylori* Infection

Nine samples were sequenced using the DNBSEQ platform, which yielded an average of 6.69 G data per sample. The average rate to the genome was 98.75%, whereas alignment to the gene sets was 68.30%. A total of 18,518 genes were detected, with variation in raw data quality Q20 ≥ 99.02 and Q30 ≥ 94.54, indicating high‐quality data.

Differential gene expression analysis revealed 76 differentially expressed genes (DEGs) when comparing the *H. pylori*‐infected and normal control groups, with 13 upregulated and downregulated genes. When comparing the TCM treatment group to the *H. pylori*‐infected group, 1500 DEGs were identified, including 1159 upregulated and 341 downregulated genes. Differential expression analysis visualized key patterns, including a Venn diagram that depicts DEG overlaps between treatment/model groups (Figure 2(a)), boxplots that display expression distribution (Figure 2(b)), hierarchical clustering of 20 shared DEGs (Figure 2(c)), and a volcano plot of DEG significance (Figure 2(d)).

FIGURE 2Transcriptome sequencing of gastric tonic treatment for *H. pylori* infection. (a) Venn diagram of differentially expressed genes. (b) Boxplot of expression levels. (c) Heatmap of clustered differentially expressed genes. (d) Volcano plot of differentially expressed genes. (e) Bubble plot of KEGG enrichment pathway analysis. (f) Bar chart of GO enrichment analysis.

**Figure:**
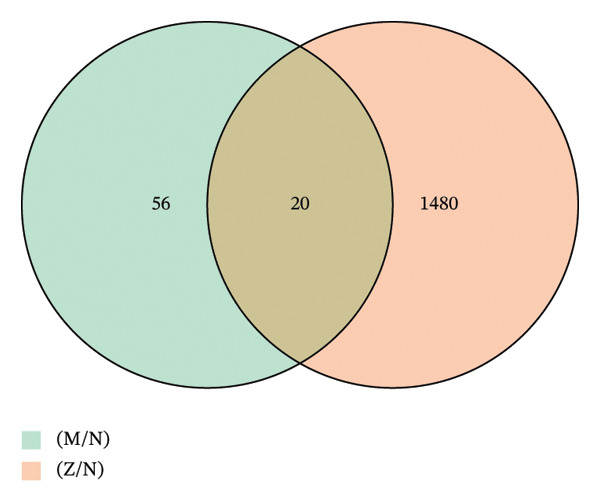
(a)

**Figure:**
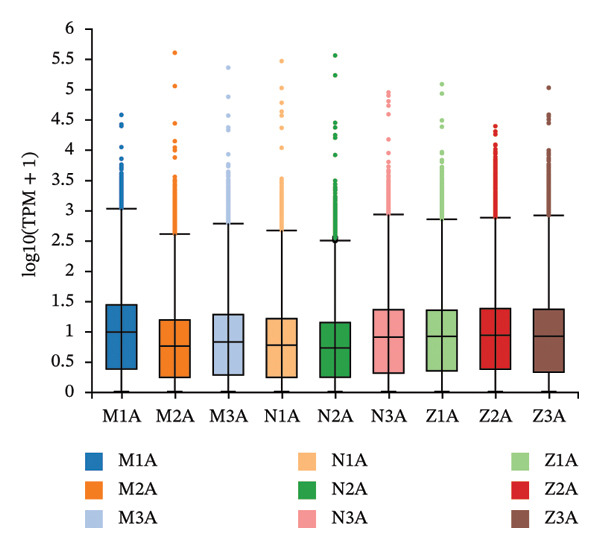
(b)

**Figure:**
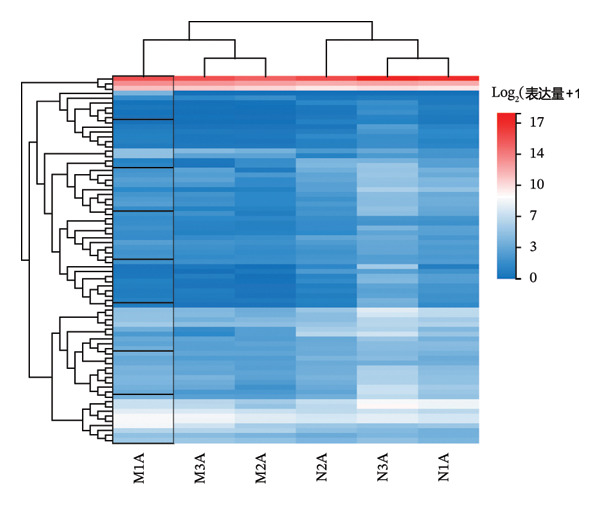
(c)

**Figure:**
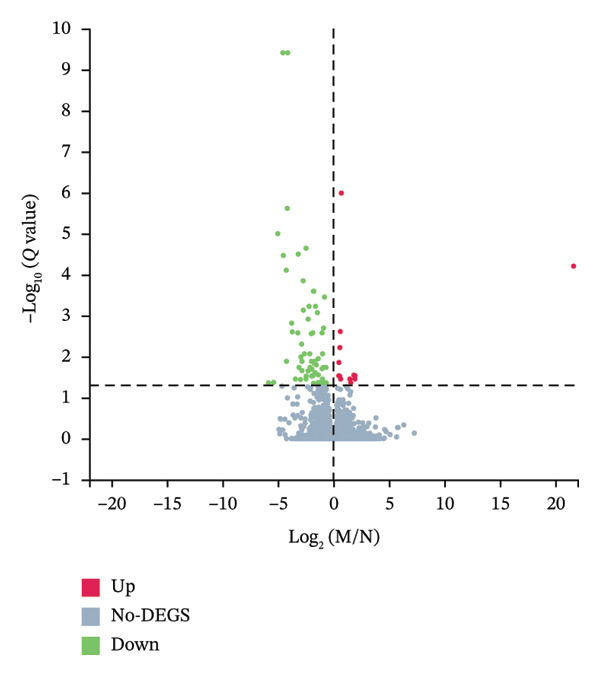
(d)

**Figure:**
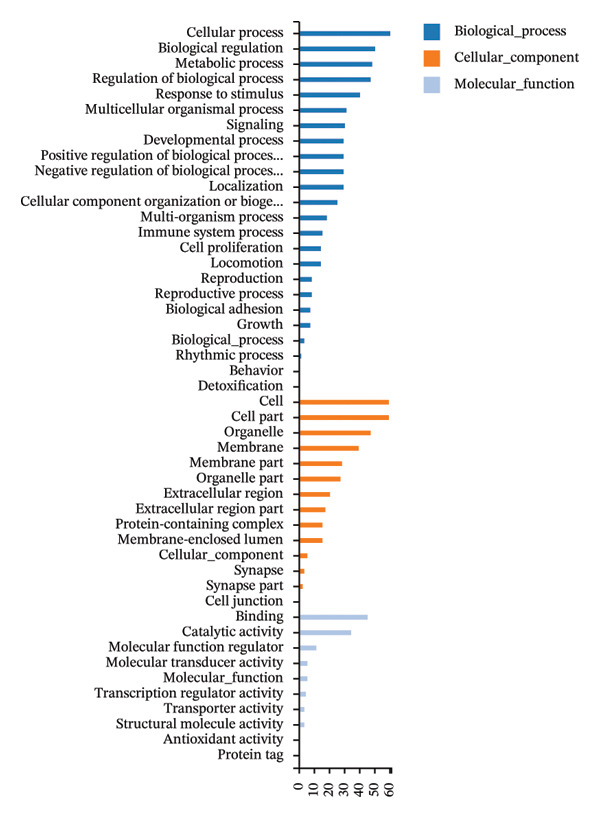
(e)

**Figure:**
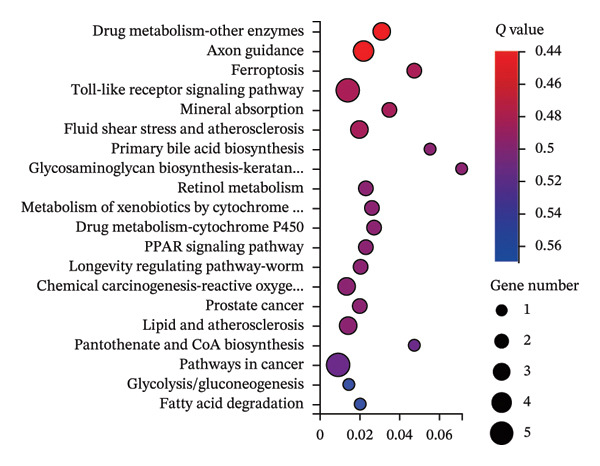
(f)

Biological functions of the differentially expressed mRNAs were explored through GO and KEGG analyses, which displayed the top 30 pathways (Figures 2(e) and 2(f)). GO highlighted pathways associated with immune responses, cell signaling, and tissue development, including responses to viruses, positive regulation of interferon‐beta production, and negative regulation of viral genome replication. Key genes, including Irf1, Hmgb1, Isg15, Fcgbp, Hsp90aa1, Oasl, Oasl2, Rsad2, and ox2 were enriched, indicating their potential regulatory roles in the immune response to *H. pylori* infection after treatment with the KaiWei JianPI Ointment.

KEGG enrichment analysis revealed mRNA involvement in drug metabolism, axon guidance, ferroptosis, fluid shear stress, atherosclerosis, primary bile acid biosynthesis, TLR signaling, PPAR signaling, and cancer pathways. These pathways are critical for the occurrence and development of gastrointestinal disease. Overall, the mechanism of action of KaiWei JianPI Ointment in combination with quadruple therapy for *H. pylori* infection may be linked to genes involved in ferroptosis and TLR signaling, warranting further research.

### 3.2. Establishment of *H. pylori* Infection Model in SD Rats

Gastric mucosal tissues from rats were embedded in paraffin, sectioned, and observed by Warthin–Starry silver staining (Figure 3(b)). We observed that in the gastric mucosa of the *H. pylori*‐infected group, silver ions associated with *H. pylori* were reduced and appeared brown or black, exhibiting predominantly curved, short rod, or spiral shapes. In contrast, *H. pylori* colonization was not observed in the gastric tissues of the control group.

FIGURE 3Verification of the *H. pylori* infection model in SD rats. Representative images of gastric mucosal tissues from the uninfected and Hp‐infected groups using different staining methods. (a) IHC staining showing brownish‐positive *H. pylori* antigen distribution; (b) Warthin–Starry silver staining showing brown/black spiral bacteria; (c) Giemsa staining displaying purplish‐red *H. pylori* with typical S‐shaped morphology. (d) H&E staining illustrating bacterial colonization (bluish‐purple) accompanied by inflammatory infiltration. No bacteria were observed in the control group. Scale bars: 400 μm (10×), 100 μm (20×), and 50 μm (100×).

**Figure:**
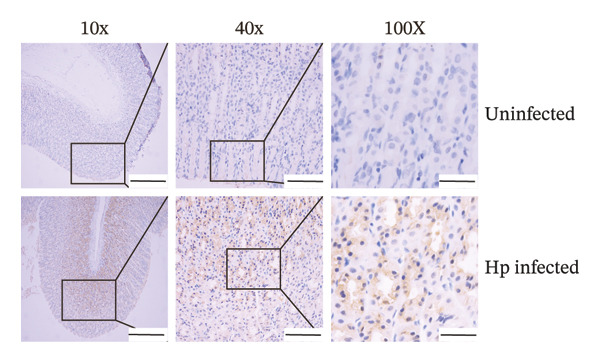
(a)

**Figure:**
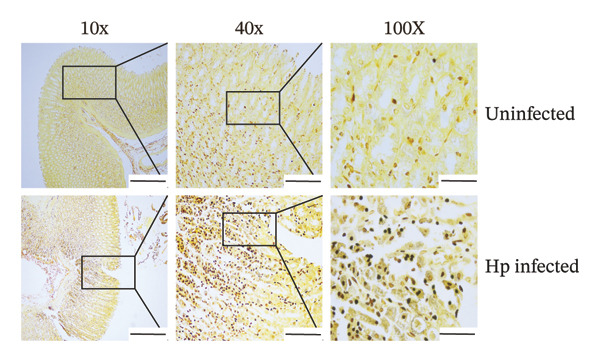
(b)

**Figure:**
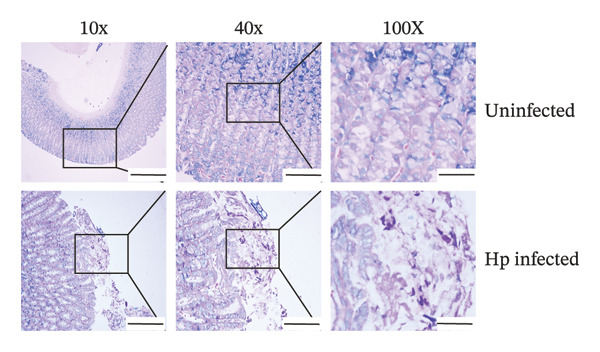
(c)

**Figure:**
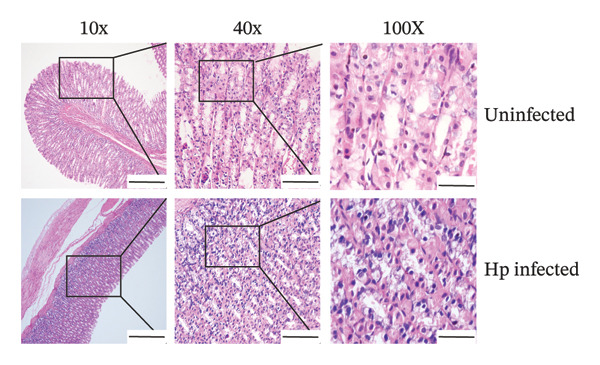
(d)

*H. pylori* and its components exhibited brownish‐positive immunohistochemical staining (Figure 3(a)). Clusters of rod‐shaped and microspiral S‐shaped *H. pylori* were observed in the surface epithelium, glandular lumen, and gastric pits of the infected gastric mucosa. Giemsa staining (Figure 3(c)) showed purplish‐red staining, with typical S‐shaped or seagull‐shaped curves located on the mucosal surface and in deeper gastric pits and junctions of the epithelial cells.

H&E staining (Figure 3(d)) indicated bluish‐purple bacteria and background, primarily concentrated within the gastric pits or glandular lumens on the gastric surface, displaying curved punctate or small‐dot patterns accompanied by significant inflammatory cell infiltration and pathological changes in the gastric mucosa. Consistent results with the four staining methods confirmed the successful establishment of *H. pylori* infection model in SD rats.

### 3.3. Ferroptosis Inhibition in *H. pylori* Infection by KaiWei JianPI Ointment

After euthanizing the rats, fresh gastric mucosal tissues were collected, and Fe^2+^ concentration was measured using a colorimetric assay. Results (Figure 4(a)) indicated that Fe^2+^ levels were significantly elevated in the gastric mucosa of the *H. pylori*‐infected group compared to that in the control, whereas the combination treatment group showed a notable decrease in Fe^2+^ levels (*p* < 0.05).

FIGURE 4Inhibition of *H. pylori* infection‐induced ferroptosis by gastric tonic. (a–c) Levels of Fe^2+^, MDA, and GSH in gastric mucosal tissues from the uninfected, *H. pylori* infected, and treatment groups. (d) Quantitative analysis of GPX4 protein expression relative to β‐actin. (e) Representative Western blot images of GPX4 in gastric mucosal tissues. Data are mean ± SD, *n* = 3. ^∗^ *p* < 0.05, ^∗∗^ *p* < 0.01, ^∗∗∗^ *p* < 0.001; ^ns^: *p* > 0.05.

**Figure:**
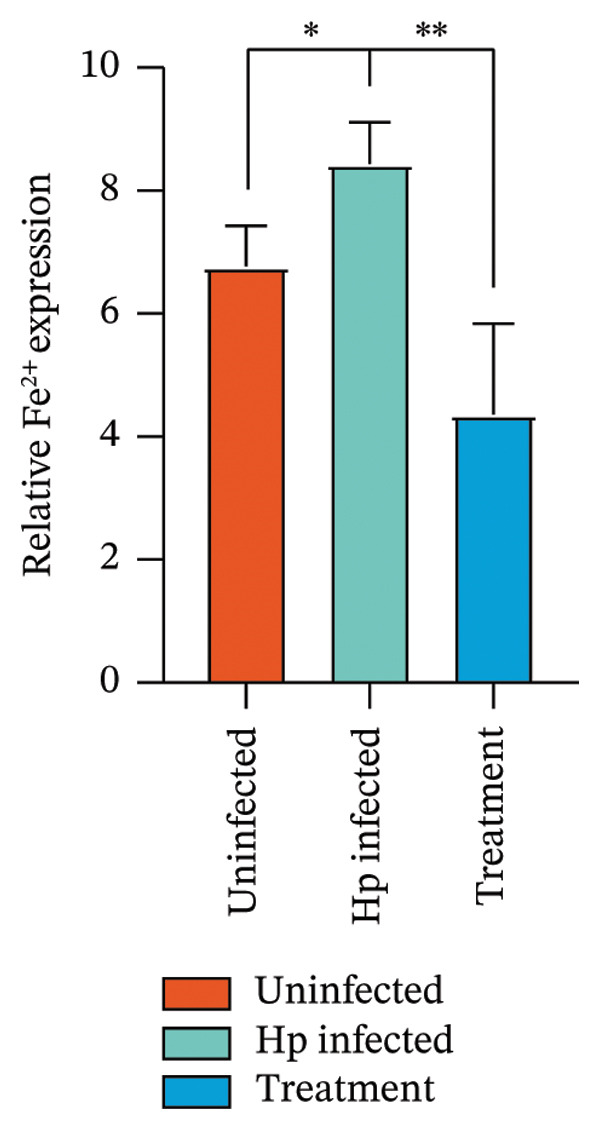
(a)

**Figure:**
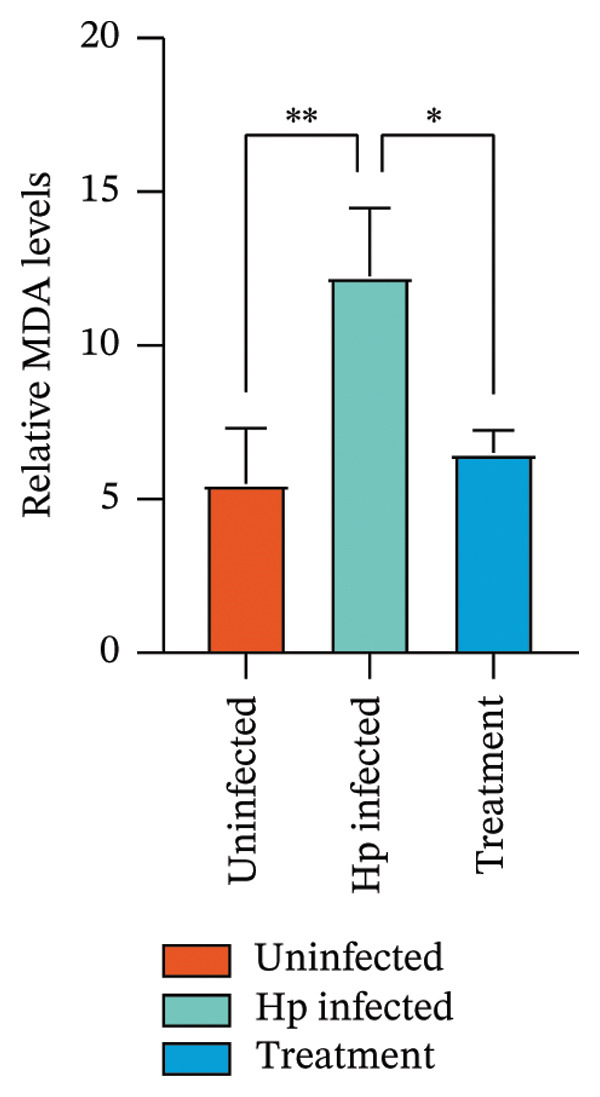
(b)

**Figure:**
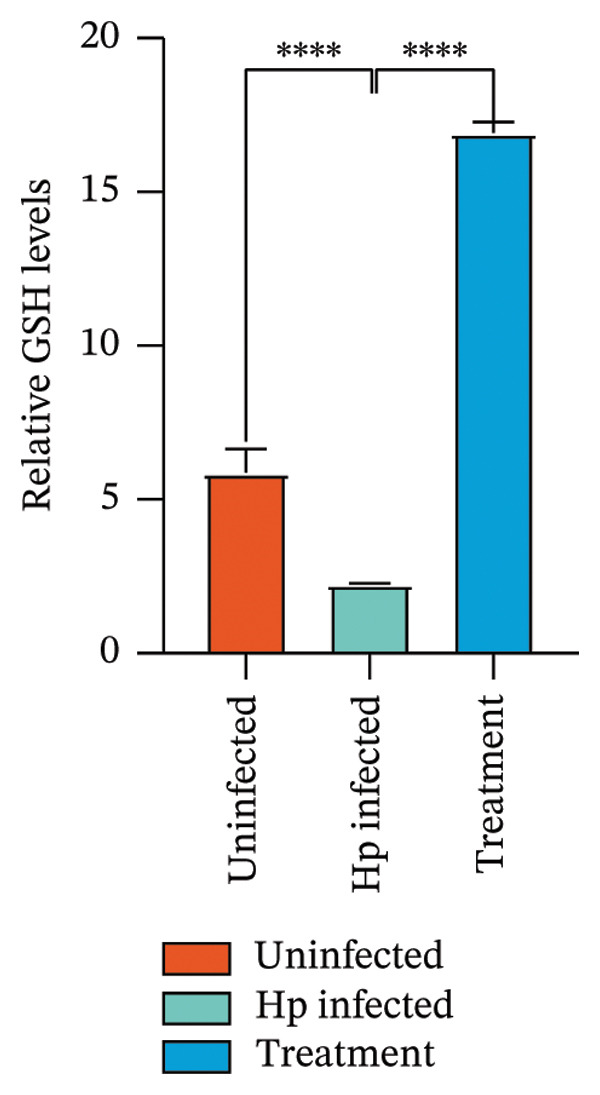
(c)

**Figure:**
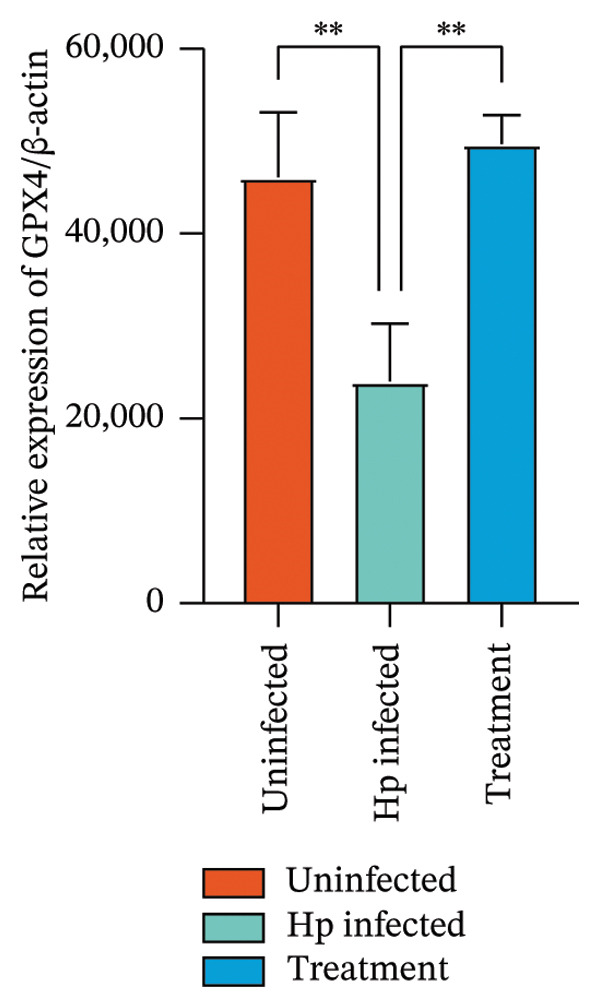
(d)

**Figure:**
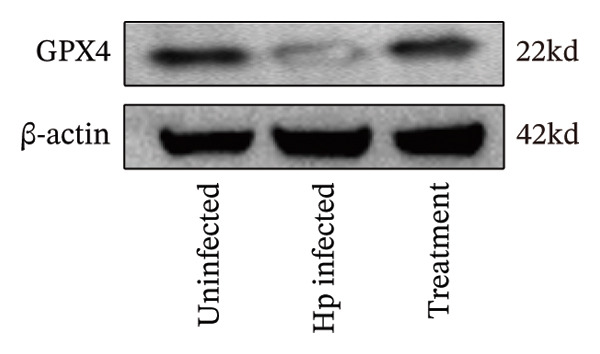
(e)

To validate these findings, MDA in the gastric mucosal tissues of rats was assessed using a lipid peroxidation assay kit. MDA levels were significantly higher in the *H. pylori*‐infected group than in the uninfected group, whereas the combination treatment group showed a significant reduction (Figure 4(b)). Additionally, in contrast to the depletion observed in the model group, GSH levels were significantly increased in the combination treatment group (*p* < 0.05, Figure 4(c)).

Western blot analysis of ferroptosis‐related proteins demonstrated downregulation of GPX4 expression in the gastric mucosa of *H. pylori*‐infected rats, whereas the combination treatment group showed upregulation (Figures 4(d) and 4(e)). Mechanistically, the inhibition of ferroptosis was evidenced by a dual effect: the suppression of proferroptotic drivers (Fe^2+^ and MDA) and the boosting of antioxidant capacity. Specifically, the treatment lowered oxidative stress while concurrently elevating the levels of GSH and GPX4, thereby protecting gastric mucosal cells from iron‐dependent oxidative death.

### 3.4. KaiWei JianPI Ointment Alleviates Ferroptosis in *H. pylori* Infection Through Fcgbp‐HMGB1/TLR4 Signaling Pathway

Transcriptome sequencing identified DEGs enriched in the HMGB1/TLR4 and ferroptosis signaling pathways. To determine whether the KaiWei JianPI Ointment combined with quadruple therapy influenced Fcgbp expression and HMGB1/TLR4 signaling, a Western blot of Fcgbp protein levels was performed in the rat gastric mucosal tissues. Results indicated that Fcgbp protein expression was upregulated in the *H. pylori*‐infected group compared to the uninfected group, whereas it was downregulated in the combination treatment group (Figure 5(a)).

FIGURE 5Regulatory effect of KaiWei JianPI ointment on the TLR4 signaling pathway and Fcgbp expression. (a–c) Relative mRNA expression levels of Fcgbp, HMGB1, and TLR4 in gastric mucosal tissues from the uninfected, Hp infected, and treatment groups, determined by qRT‐PCR. (d) Representative western blot images of Fcgbp, HMGB1, and TLR4 proteins. β‐actin was used as a loading control. (e–g) Quantitative analysis of the relative protein expression levels of Fcgbp, HMGB1, and TLR4. Data are presented as mean ± SD (*n* = 3 per group). ^∗^ *p* < 0.05, ^∗∗^ *p* < 0.01, ^∗∗∗^ *p* < 0.001; ^ns^: *p* > 0.05.

**Figure:**
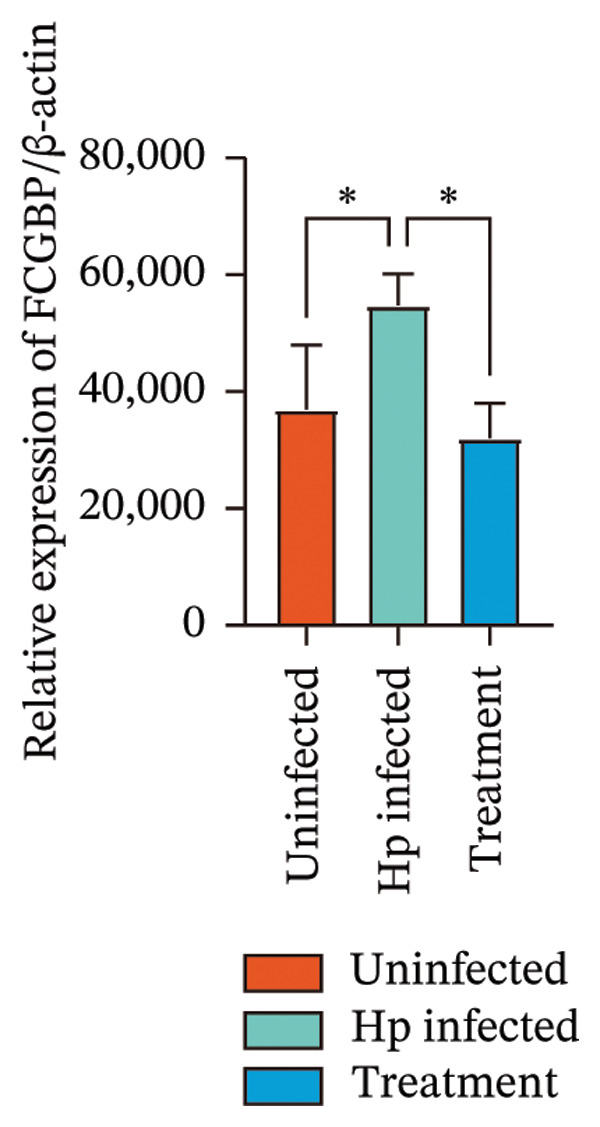
(a)

**Figure:**
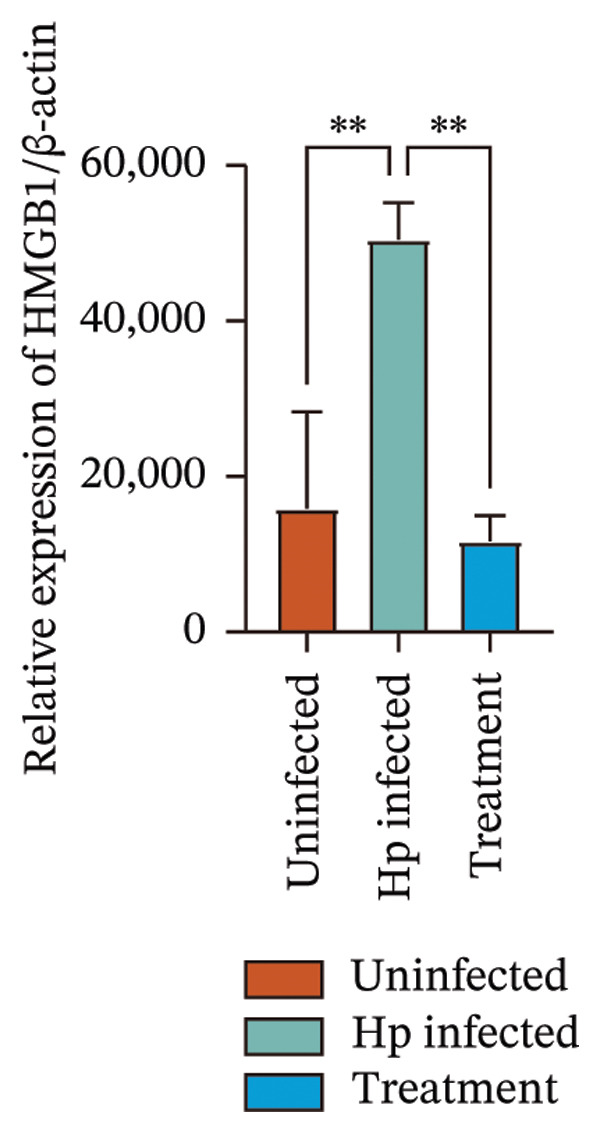
(b)

**Figure:**
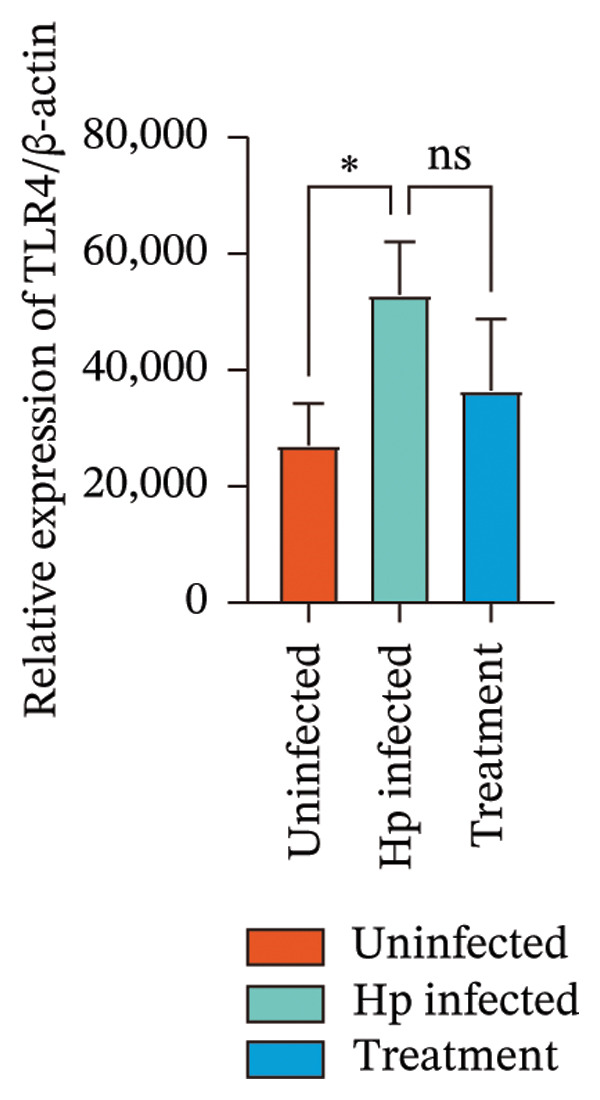
(c)

**Figure:**
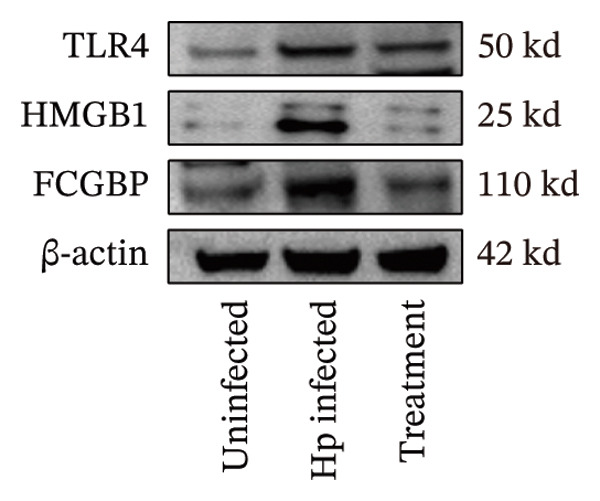
(d)

**Figure:**
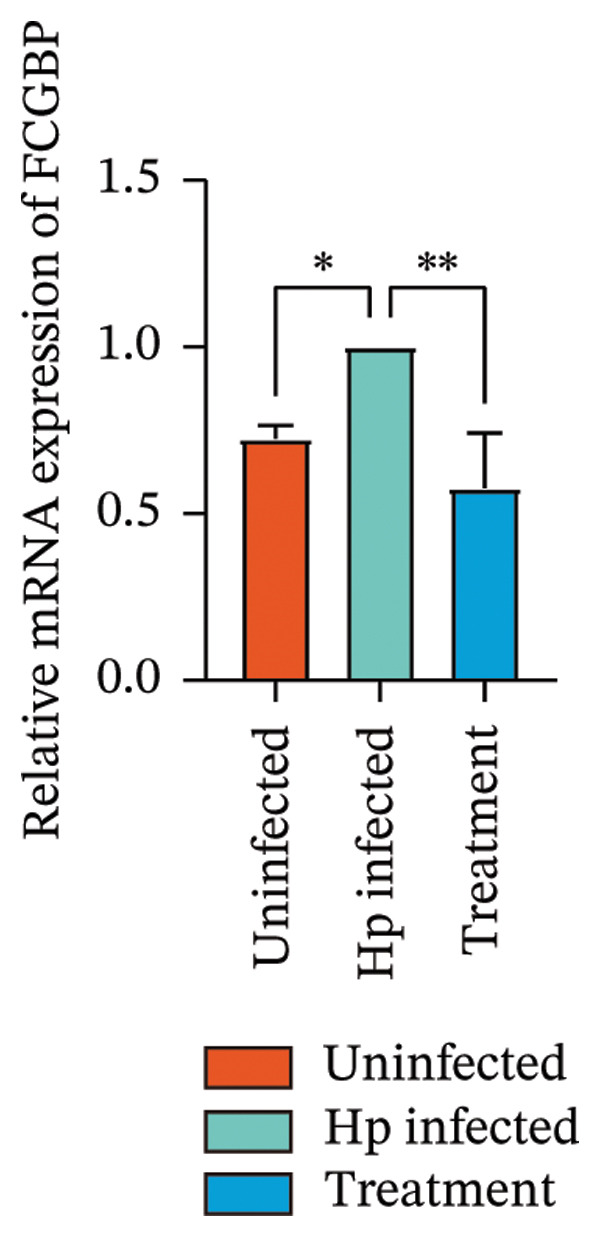
(e)

**Figure:**
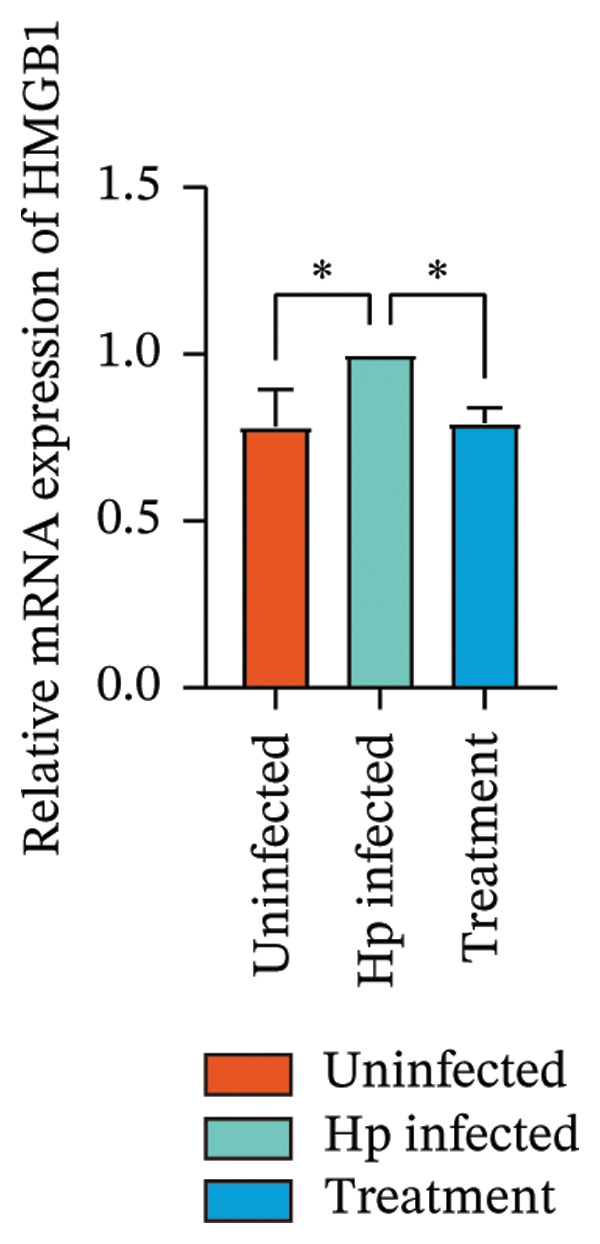
(f)

**Figure:**
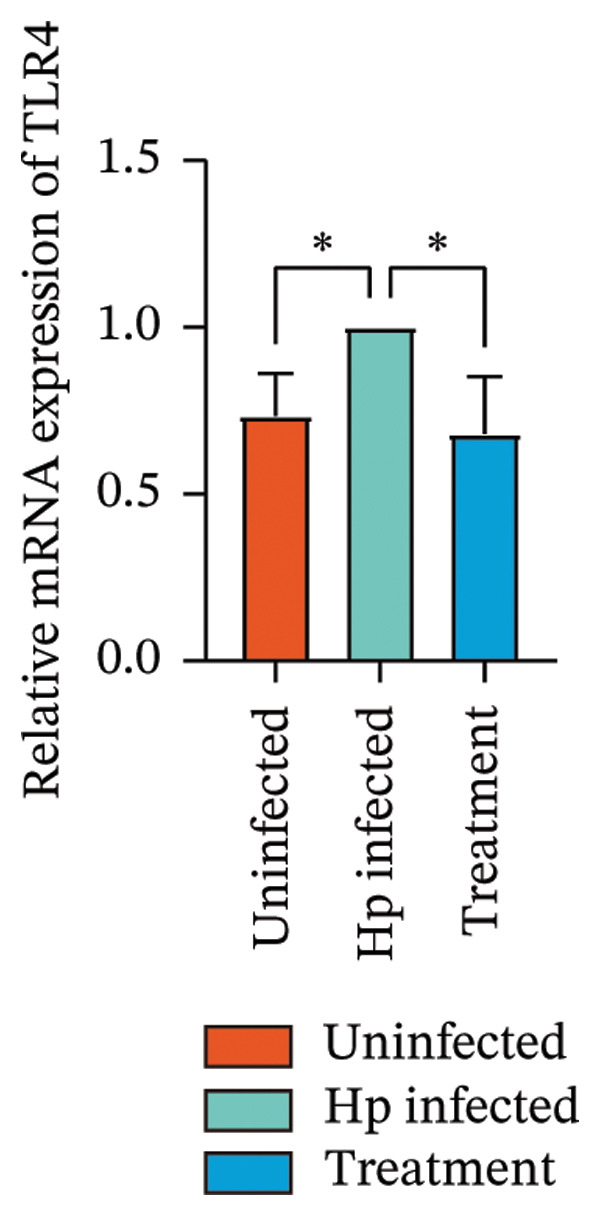
(g)

Fcgbp mRNA expression was significantly elevated in the *H. pylori*‐infected group and significantly decreased in the combination treatment group (Figures 5(d) and 5(e)), suggesting that KaiWei JianPI Ointment combined with quadruple therapy downregulates Fcgbp expression. Previous studies indicate that HMGB1/TLR4 signaling not only regulates ferroptosis but also serves as a downstream effector, inducing inflammation related to ferroptosis. HMGB1/TLR4 pathway protein expression in gastric mucosal tissues (Figures 5(b), 5(c), and 5(d)) was upregulated in the *H. pylori*‐infected group compared to that in the uninfected group and downregulated in the combination treatment group (*p* < 0.05).

Subsequently, qPCR analysis showed elevated expression of pathway‐related mRNA in the *H. pylori*‐infected group compared to that in the uninfected group and decreased expression in the combination treatment group (Figures 5(e), 5(f), and 5(g)). These findings suggest that the KaiWei JianPI Ointment inhibits ferroptosis during *H. pylori* infection by activating the HMGB1/TLR4 pathway. Overall, we propose that the KaiWei JianPI Ointment downregulates Fcgbp to modulate HMGB1/TLR4 signaling, providing a potential therapeutic strategy against ferroptosis in *H. pylori* infection; however, further investigation into the mechanisms by which Fcgbp regulates this pathway is warranted.

## 4. Discussion

**FIGURE 6:**
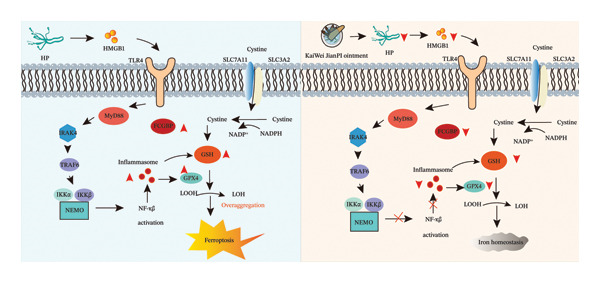
Mechanistic pathway of HMGB1/TLR4 signaling and ferroptosis modulation by KaiWei JianPI Ointment in *H. pylori* infection. HMGB1 is released from damaged gastric epithelial cells and binds to TLR4 on the cell surface, leading to TLR4 activation. This triggers a signaling cascade involving MyD88 and TRIF, resulting in the activation of NF‐κB and the release of proinflammatory cytokines. The inflammatory environment increases intracellular levels of Fe^2+^ and lipid peroxides, promoting ferroptosis in gastric epithelial cells. Abbreviations: Hp: *Helicobacter pylori*; HMGB1: high‐mobility group box 1; TLR4: toll‐like receptor 4; Fcgbp: Fc fragment of IgG binding protein; NF‐κB: nuclear factor kappa‐light‐chain‐enhancer of activated B cells; GSH: reduced glutathione; LOOH: lipid hydroperoxide; GPX4: glutathione peroxidase 4.

*Helicobacter pylori* infection continues to present a major global public health challenge, acting as the primary etiological agent for gastritis, peptic ulcers, and gastric cancer [19]. While standard therapies remain the cornerstones of management, their efficacy is increasingly compromised by the alarming rise in antibiotic resistance, which often leads to persistent infection and chronic mucosal remodeling [20]. In this study, we investigated the therapeutic mechanisms of the KaiWei JianPI Ointment against *H. pylori*, specifically focusing on its regulatory influence on ferroptosis and HMGB1/TLR4 signaling (Figure 6). Our findings suggest that this TCM formulation may provide a novel approach to mitigating *H. pylori*‐associated pathologies, particularly in the context of failing antibiotic regimens.

Through network pharmacology, we identified 152 active components within the ointment that interact with key proteins governing immune response and cellular signaling pathways. This multitarget profile suggests a broad mechanism of action, which was reflected in the transcriptional changes observed in gastric tissue. The analysis revealed a substantial number of DEGs, underscoring the ointment’s multifaceted effects. Specifically, among these, 76 DEGs were linked to immune response pathways, implying a capacity to enhance the host’s defense against *H. pylori*. This bioinformatic evidence is supported by established modern pharmacological studies on two core components identified in our screening: kaempferol and quercetin.

Kaempferol exerts antibacterial effects through both direct and indirect mechanisms. It targets the Cagβ subunit of the T4SS secretion system, inhibiting the translocation of the CagA virulence protein into host cells, while also disrupting biofilm formation and reducing extracellular DNA release [21]. Furthermore, it downregulates the expression and secretion of proinflammatory mediators (TNF‐α, IL‐1β, and IL‐8) in AGS cells, thereby attenuating the inflammatory cascade. Supporting its translational potential, recent research indicates that flavonoid‐antibiotic co‐crystallization strategies can significantly enhance conventional antibiotic efficacy [22]. Similarly, quercetin exhibits anti‐*H. pylori* activity via multiple pathways. Its molecular structure allows it to inhibit bacterial urease by forming stable hydrogen bonds and to bind the Ddl enzyme, disrupting cell wall synthesis. It also interferes with bacterial transcriptional regulation by complexing with the HsrA protein [23]. At the host level, quercetin modulates the p38 MAPK signaling pathway, balances BCL‐2/BAX expression, and suppresses infection‐induced abnormal apoptosis in gastric epithelial cells [24]. However, as its direct bactericidal activity is moderate, future research into advanced delivery systems may be necessary to fully exploit its therapeutic potential.

Emerging evidence underscores the pivotal role of ferroptosis, an iron‐dependent form of inflammatory cell death, in *H. pylori*‐associated gastric pathologies [25]. Infection induces ferroptosis through a multipronged strategy that initiates and exacerbates MDA. This process begins when infection‐activated neutrophils generate excessive reactive oxygen and nitrogen species (ROS/RNS), attacking polyunsaturated fatty acids (PUFAs) in cell membranes [12, 26]. Oxidative damage is further amplified through the disruption of iron metabolism; *H. pylori* upregulates hepcidin via the IL‐6/STAT3 pathway, leading to dysregulated iron absorption and an elevated intracellular labile iron pool [27, 28]. Complicating this landscape, the bacterium simultaneously modulates the host environment to ensure its own survival; for instance, secreted outer membrane vesicles (OMVs) can downregulate the iron importer TFRC/TFR1 while upregulating the cystine transporter SLC3A2, inhibiting ferroptosis to establish a survivable niche [29].

*Mechanistically, H. pylori* activates gastric mucosal cells to release HMGB1. As a damage‐associated molecular pattern (DAMP), HMGB1 binds to TLR 4 (TLR4), triggering the NF‐κB pathway [30, 31]. This activation not only drives a robust proinflammatory cascade but is also mechanistically linked to the induction of ferroptosis in gastric epithelial cells, thereby exacerbating tissue damage and fostering a procarcinogenic microenvironment [32].

Our study demonstrates that the KaiWei JianPI Ointment confers protection by strategically intervening in this pathological axis. Central to our observed mechanism is the ointment’s significant downregulation of Fcgbp in *H. pylori*‐infected gastric tissue. We propose that this downregulation serves as a crucial upstream event. By attenuating Fcgbp expression, the formulation appears to temper the excessive activation of the HMGB1/TLR4 signaling axis, as evidenced by the suppression of key pathway components in our experimental model. This targeted modulation likely contributes to inhibiting ferroptosis, a hypothesis corroborated by our in vivo results showing reduced accumulation of Fe^2+^ and MDA. In conclusion, our preliminary findings suggest a potential mechanism wherein the KaiWei JianPI Ointment, via its multicomponent composition, may associate with the downregulation of Fcgbp. This action appears to contribute to the inhibition of the overactive HMGB1/TLR4 pathway, thereby potentially mitigating ferroptotic cell death. While further validation is needed, this study offers initial insights into how targeting the Fcgbp/HMGB1/TLR4 axis could underlie the therapeutic efficacy of this TCM formulation against *H. pylori*‐induced gastropathy. We therefore emphasize the preliminary nature of these results and advocate for expanded cohort studies to verify this mechanism.

## 5. Conclusions

The KaiWei JianPI Ointment is a novel therapy for treating *H. pylori* infection by downregulating Fcgbp and modulating HMGB1/TLR4 signaling to inhibit ferroptosis. Besides expanding therapeutic approaches for *H. pylori* infection, this study also offers new insights into the application of TCM in modern healthcare. However, further investigations are needed to elucidate the specific mechanisms by which Fcgbp functions within the HMGB1/TLR4 signaling pathway in the context of *H. pylori* infection. Additionally, this study was limited by its relatively small sample size and reliance on animal models, rather than clinical data. Future studies should involve larger sample sizes and incorporate clinical trials to validate these results.

## Author Contributions

Caiqun Bie and Jie Liu designed this study. Animal experiments were conducted by Xiaoying Zhu, Yubo Jin, and Huijun Tang. Relevant information for this study was collated by Meng Chen and Yan Dang. The manuscript was initially drafted by Xiaoying Zhu, and subsequently revised and polished by Caiqun Bie and Xianfeng Qin. Xiaoying Zhu and Jie Liu reformatted all materials included in this study and submitted them for consideration. All authors accept responsibility for the integrity and accuracy of this work.

## Funding

This study was supported by the Shenzhen Association of Traditional Chinese Medicine Fund (2024052F), the Scientific Research Project of Guangdong Provincial Bureau of Traditional Chinese Medicine (202405112001157150), and the National Key Research and Development Program of the National Natural Science Foundation of the China Youth Science Fund Project (82305236).

## Conflicts of Interest

The authors declare no conflicts of interest.

## Data Availability

The data that support the findings of this study are available on request from the corresponding author. The data are not publicly available due to privacy or ethical restrictions.
